# Gut microbiota and metabolite interface-mediated hepatic inflammation

**DOI:** 10.1097/IN9.0000000000000037

**Published:** 2024-01-25

**Authors:** Ming Yang, Katina Massad, Eric T. Kimchi, Kevin F. Staveley-O’Carroll, Guangfu Li

**Affiliations:** 1 Department of Surgery, University of Missouri, Columbia, MO, USA; 2 NextGen Precision Health Institute, University of Missouri, Columbia, MO, USA; 3 Harry S. Truman Memorial VA Hospital, Columbia, MO, USA; 4 Ellis Fischel Cancer Center, University of Missouri, Columbia, MO, USA; 5 Department of Molecular Microbiology and Immunology, University of Missouri, Columbia, MO, USA

**Keywords:** liver inflammation, gut microbiota, metabolite, metabolic regulation, chronic liver disease, clinical trials

## Abstract

Immunologic and metabolic signals regulated by gut microbiota and relevant metabolites mediate bidirectional interaction between the gut and liver. Gut microbiota dysbiosis, due to diet, lifestyle, bile acids, and genetic and environmental factors, can advance the progression of chronic liver disease. Commensal gut bacteria have both pro- and anti-inflammatory effects depending on their species and relative abundance in the intestine. Components and metabolites derived from gut microbiota–diet interaction can regulate hepatic innate and adaptive immune cells, as well as liver parenchymal cells, significantly impacting liver inflammation. In this mini review, recent findings of specific bacterial species and metabolites with functions in regulating liver inflammation are first reviewed. In addition, socioeconomic and environmental factors, hormones, and genetics that shape the profile of gut microbiota and microbial metabolites and components with the function of priming or dampening liver inflammation are discussed. Finally, current clinical trials evaluating the factors that manipulate gut microbiota to treat liver inflammation and chronic liver disease are reviewed. Overall, the discussion of microbial and metabolic mediators contributing to liver inflammation will help direct our future studies on liver disease.

## 1. Introduction

Liver inflammation accompanies most chronic liver diseases, such as alcoholic liver disease (ALD) and non-alcoholic fatty liver disease (NAFLD), accelerating their progression to liver fibrosis, cirrhosis, and primary liver cancers ^[1,2]^. The term metabolic dysfunction-associated fatty liver disease (MAFLD) has been suggested to replace NAFLD recently, considering the criteria for disease diagnosis and heterogeneity of risk factors ^[3,4]^. Both NAFLD and MAFLD in this review are used to keep consistent with the literature reports. Factors contributing to liver injury, such as alcohol consumption, pathogenic infection, and diet, can regulate liver inflammation. The gut–liver axis, a physiological crosstalk between the gut and liver via immunological and metabolic signals, can be regulated by dietary, genetic, epigenetic, and environmental factors ^[5,6]^. Most metabolic diseases, such as obesity ^[7,8]^, type 2 diabetes ^[9,10]^, and cardiovascular diseases (CVDs) ^[11,12]^, are associated with a disruption of the balance of gut microbiota (dysbiosis) leading to changes in intestinal permeability and systemic inflammation ^[13]^. Accumulating evidence also shows that gut microbiota dysbiosis can specifically impact hepatic inflammation and metabolism in chronic liver diseases at different stages ^[14,15]^.

Hepatic parenchymal and non-parenchymal cells orchestrate liver inflammation, which, in turn, are also impacted by secreted inflammatory cytokines and chemokines from inflammatory immune cells. Hepatocytes account for 80% of liver parenchymal cells. Pattern recognition receptors (PRRs) expressed by hepatocytes can be activated by microbial-associated molecular patterns (MAMPs) and pathogen-associated molecular patterns (PAMPs) to produce inflammatory factors to activate liver resident cells ^[16,17]^. The activation of liver resident immune cells (eg, liver resident macrophages or Kupffer cells) and infiltration of inflammatory immune cells (eg, monocytes and neutrophils) contribute to liver inflammation. Pro-inflammatory cytokines (eg, interleukin-1β or IL-1β and tumor necrosis factor-α or TNF-α) secreted from activated inflammatory cells cause hepatocyte injury, while the secreted chemokines (eg, monocyte chemoattractant protein-1 or MCP-1/CCL2 and C-X-C motif chemokine ligand 1) chemoattract monocytes and neutrophils into the liver, accelerating liver inflammation ^[18,19]^.

In this mini review, the roles of gut microbiota and gut microbiota-derived metabolites in the regulation of hepatic inflammation are summarized. The regulatory functions of different gut microbial genera or species in liver inflammation and the associated cellular mechanisms and molecular signaling pathways are reviewed. Finally, the potential strategies via modulating the homeostasis of gut microbiota to suppress liver inflammation are discussed, as well as the findings of current clinical trials.

## 2. The role of gut microbiota in liver inflammation

The dual roles of the gut microbiota, including both anti-inflammatory and pro-inflammatory processes, are dependent on the relative abundances of gut microbiota and their species. Alterations of gut microbiota, such as an increase of pathogenic bacteria and a decrease of beneficial bacteria, are commonly associated with liver inflammation.

### 2.1 Pathogenic bacteria in liver inflammation

Intragastric administration of pathogenic bacteria Group A *Streptococcus* and *Salmonella enteritidis* advanced concanavalin A (ConA)-induced liver hepatitis in mice by activating liver immune cells, whereas depletion of intestinal Gram-negative bacteria mediated by oral treatment of gentamycin decreased ConA-induced liver injury by suppressing natural killer T (NKT) cell activation ^[20]^. The relative abundance of *Escherichia coli* has been shown to be increased in patients with NAFLD compared with healthy controls, which can accelerate the development of hepatic steatosis, inflammation, and fibrosis. Mechanistic studies showed that flagella from *E. coli* can activate toll-like receptor 5 (TLR5) in liver sinusoidal endothelial cells (LSECs) to promote liver injury and epithelial to mesenchymal transition through the myeloid differentiation primary response gene 88 (MYD88)/Twist1 signaling pathway ^[21]^. In addition, high counts of *E. coli* were significantly and positively associated with hepatocellular carcinoma (HCC) in human patients ^[22]^. Inhibition of bile acid (BA)-converting *Clostridium* species (eg, *Clostridium scindens*) that are responsible for converting primary BAs (PBAs) to secondary BAs (SBAs) can increase the expression of CXCL16 on LSECs to attract the infiltration of hepatic CXCR6^+^ NKT cells to inhibit liver cancer progression ^[23]^. Another study showed that synergistic treatment of *Bilophila wadsworthia* promoted high-fat diet (HFD)-induced local and systemic inflammation (upregulation of interferon-gamma or IFN-γ and IL-6), intestinal barrier interruption, BA and glucose metabolism dysregulation, and hepatic steatosis. In contrast, the administration of a probiotic strain *Lactobacillus rhamnosus* CNCM I-3690 ameliorated *B. wadsworthia*-induced metabolic dysregulation and inflammation and strengthened intestinal barrier integrity ^[24]^. Overall, a high abundance of pathogenic and inflammation-promoting bacteria can increase the circulating MAMPs and PAMPs to active PRRs in liver cells, which can induce and promote diet/chemical/toxin-induced liver inflammation, injury, and fat accumulation.

### 2.2 Beneficial bacteria in liver inflammation

Patients with severe alcoholic hepatitis and prednisolone who received fecal microbial transplantation (FMT) from healthy donors through a naso-duodenal tube had a 90-day survival rate of 75% compared with 56% in the control group with only prednisolone ^[25]^. FMT treatment reduced pathogenic bacteria such as *Campylobacter* (a microaerophilic bacterium) and anaerobic bacteria *Parcubacteria*, *Weisella*, and *Leuconostocaceae*, while increasing the abundance of *Alphaproteobacteria* and *Thaumarchaeota* ^[25]^.

Administration of *Akkermansia muciniphila*, a mucin-degrading bacterium, decreased HFD-induced body weight gain, hepatic steatosis, and liver injury in pathogen-free (SPF) male C57BL/6 mice. The hepatic protective effects of *A. muciniphila* were associated with the alteration of gut microbiota, including a decrease in the abundance of commensal bacteria *Alistipes*, *Blautia, Butyricimonas*, *Lactobacilli*, and *Tyzzerella* and an increase of potential beneficial bacteria *Allobaculum*, *Anaeroplasma*, *Osclibacter*, *Ruminiclostridium*, and *Rikenella* ^[26]^.

*Bifidobacterium pseudolongum* (strain CCFM1253) pre-intervention significantly reduced lipopolysaccharide (LPS)-induced acute liver injury in mice by decreasing serum levels of alanine transaminase (ALT) and aspartate aminotransferase (AST) ^[27]^. In addition, *B. pseudolongum* (strain CCFM1253) pre-intervention dampened liver inflammation by decreasing the expression of pro-inflammatory cytokines such as TNF-α, IL-1β, and IL-6 and reduced oxidative stress by increasing activities of anti-oxidative enzymes such as superoxide dismutase (SOD), catalase, and glutathione peroxidase ^[27]^. For the gut microbial profile, *B. pseudolongum* (strain CCFM1253) pre-intervention increased the relative abundance of *Alistipes* and *Bifidobacterium* and decreased the proportion of uncultured *Bacteroidales*, *Muribaculum*, *Parasutterella*, and *Ruminococcaceae* UCG-010 ^[27]^.

Treatment of *Lactobacillus gasseri* strain CKCC1913 in diabetic mice reduced insulin resistance, fasting blood glucose levels, serum levels of pro-inflammatory cytokines (eg, TNF-α and IL-6), and hepatic oxidative stress-induced damage by increasing SOD activity and decreasing malondialdehyde levels. This therapy increased the abundance of beneficial bacteria such as *Parabacteroides merdae* and the production of short-chain fatty acids (SCFAs) to reduce liver oxidative stress and inflammation ^[28]^. In vitro treatment of *Lactobacillus sakei* MJM60958 inhibited oleic acid and cholesterol-induced hepatocyte lipid accumulation. Additionally, in vivo administration of *L. sakei* MJM60958 suppressed HFD-induced NAFLD features in mice, including significant reduction of liver weight, body weight, and blood levels of ALT, AST, triglyceride (TG), urea nitrogen, and uric acid ^[29]^. Treatment with human commensal *L. rhamnosus* GG5 protected against acetaminophen overdose and acute alcohol consumption–induced liver oxidative injury by producing 5-methoxyindoleacetic acid to activate nuclear factor erythroid 2–related factor 2 transcription factor ^[30]^.

Ethanol feeding can induce gut dysbiosis by increasing the permeability of the gut barrier, relative abundance of pathogenic *Escherichia* and *Staphylococcus*, and liver inflammation, while depleting SCFA-producing bacteria, such as *Prevotella*, *Faecalibacterium*, and *Clostridium* ^[31]^. In contrast, *Pediococcus pentosaceus* (strain CGMCC 7049) administration reduced ethanol-induced liver injury by decreasing serum ALT, AST, and TG levels, neutrophil infiltration, and the expression of inflammatory cytokines such as TNF-α and chemokines such as CCL2 and macrophage inflammatory protein-1α (MIP-1α/CCL3). In addition, *P. pentosaceus* treatment increased the expression of tight junction protein ZO-1, mucin proteins, and antimicrobial peptide Reg3β in the intestine. The relative abundance of SCFA-producing gut bacteria was also restored with *P. pentosaceus* administration, increasing propionic acid and butyric acid levels.

Furthermore, combined treatment with species such as *Lactobacillus lactis* and *P. pentosaceus* can reduce NAFLD activity score, improve gut-tight junction, alter gut microbial profiles (eg, *Firmicutes*/*Bacteroidetes* ratio), and restore the important metabolites such as SCFAs, BAs, and tryptophan metabolites to reduce systemic and locally liver and intestinal inflammation ^[32,33]^. In summary, gut microbiota plays dual roles in liver inflammation, depending on bacterial species and their abundances (Table 1).

**Table 1:** Functions of bacterial supplementation in liver disease.

Liver disease	Treatment	Effects	References
Concanavalin A (ConA)-induced liver hepatitis	Intragastric administration of pathogenic bacteria Group A *Streptococcus* and *Salmonella enteritidis*	Aggregation of ConA-induced liver hepatitis via activating liver natural killer T (NKT) cell activation in mice.	Chen et al ^[20]^
Non-alcoholic fatty liver disease (NAFLD)	*Escherichia coli*	*E. coli* has been shown to accelerate the development of hepatic steatosis, inflammation, fibrosis, and primary liver cancer.	Shen et al ^[21]^; Grąt et al ^[22]^
Hepatocellular carcinoma	*Clostridium species such as Clostridium scindens*	Inhibition of bile acid (BA)-converting *Clostridium* species that are responsible for converting primary BAs to secondary BAs can increase the expression of CXCL16 on LSECs to attract the infiltration of hepatic CXCR6^+^ NKT cells to inhibit liver cancer progression.	Ma et al ^[23]^
Hepatic steatosis and systemic inflammation	*Bilophila wadsworthia*	Promoting high-fat diet (HFD)-induced local and systemic inflammation, intestinal barrier interruption, BA, and dysregulation of glucose metabolism, and hepatic steatosis, which can be inhibited by treatment of a probiotic strain *Lactobacillus rhamnosus.*	Natividad et al ^[24]^
Severe alcoholic hepatitis	Fecal microbial transplantation (FMT)	Alteration of gut microbiota: *Campylobacter* and anaerobic bacteria *Parcubacteria*, *Weisella*, and *Leuconostocaceae* (↓). *Alphaproteobacteria* and *Thaumarchaeota* (↑).	Pande et al ^[25]^
Metabolic-associated fatty liver disease (MAFLD)	*Akkermansia muciniphila* administration	Alteration of gut microbiota: *Alistipes*, *Blautia, Butyricimonas*, *Lactobacilli*, and *Tyzzerella* (↓). *Allobaculum*, *Anaeroplasma*, *Osclibacter*, *Ruminiclostridium*, and *Rikenella* (↑).	Wu et al ^[26]^
LPS-induced acute liver injury	*Bifidobacterium pseudolongum* (strain CCFM1253) pre-intervention	Alteration of gut microbiota: *Bacteroidales bacterium*, *Muribaculum*, *Parasutterella* and *Ruminococcaceae* UCG-010 (↓). *Alistipes* and *Bifidobacterium* (↑).	Guo et al ^[27]^
Type 2 diabetes mellitus (T2DM)-associated liver damage	*Lactobacillus gasseri* (stain CKCC1913) treatment	*Parabacteroides merdae* (↑).	Jiang et al ^[28]^
NAFLD and hepatocyte steatosis	*In vitro* and *in vivo* treatment of *Lactobacillus sakei* MJM60958	Inhibition of oleic acid and cholesterol-induced hepatocyte lipid accumulation in vitro and suppression of HFD-induced NAFLD features in vivo.	Nguyen et al ^[29]^
Acetaminophen overdose and acute alcohol consumption–induced liver oxidative injury	Human commensal *L. rhamnosus GG5*	Produce 5-methoxyindoleacetic acid to activate nuclear factor erythroid 2–related factor 2 (Nrf2) transcription factor.	Saeedi et al ^[30]^
Alcoholic liver disease (ALD)	*Pediococcus pentosaceus* CGMCC 7049 administration	*Lactobacillus*, *Pediococcus*, *Prevotella*, *Clostridium*, and *Akkermansia* (↑).	Jiang et al ^[31^^]^
NAFLD	*Lactobacillus lactis and P. pentosaceus*	A combined treatment can reduce NAFLD activity score, improve gut-tight junction, alter gut microbial profiles (eg, *Firmicutes*/*Bacteroidetes* ratio), and restore the important metabolites such as short-chain fatty acids (SCFAs), BAs, and tryptophan metabolites to reduce systemic and locally liver and intestinal inflammation.	Yu et al ^[32]^; Lee et al ^[33]^

LPS, lipopolysaccharide.

## 3. Gut microbiota-derived metabolites regulate liver inflammation at cellular and molecular levels

Gut microbiota-derived metabolites and components have broad effects on metabolic liver diseases ^[34,35]^, accompanying liver inflammation. In this section, we review numerous gut microbial metabolites and their impact on liver inflammation at cellular and molecular levels.

### 3.1 Alcohol

Alcohol consumption is a driver for alcohol-related liver disease, which can result in the alteration of gut microbiota and increase intestinal barrier permeability ^[36,37]^. Acute alcohol use in germ-free mice significantly increased hepatic inflammation and steatosis compared with conventional mice, including upregulation of alcohol-metabolizing enzymes in the liver ^[38]^. Another murine study shows that alcohol is not metabolized by gut microbiota directly, but alcohol consumption increases the circulating acetate production to result in gut microbiota alteration in mice ^[39]^.

Endogenous ethanol is positively associated with the development of non-alcoholic steatohepatitis (NASH) in human patients with an increased abundance of alcohol-producing bacteria, such as phylum Proteobacteria, family *Enterobacteriaceae*, and genus *Escherichia* ^[40]^. Both drinking and endogenous ethanol can promote liver inflammation by regulating gut microbiota.

Both innate and adaptive immune responses play important roles in the pathogenesis of ALD. Alcohol-induced damage of the gut barrier can lead to the increase of gut microbial components such as LPS in the portal vein circulation to activate intrahepatic innate immune cells (eg, macrophages) via TLR4, which induces liver inflammation and expression of many pro-inflammatory cytokines (eg, CCL2) ^[41]^. Meanwhile, impaired cytotoxic T cells, a decrease of regulatory T cells (Tregs) and B cells, and an increase of T_H_17 cells and CD57^+^ T cells are shown in patients with alcoholic steatohepatitis ^[41–43]^.

### 3.2 Indole and tryptophan derivatives from gut microbiota

Indole is a microbial metabolite produced from the aromatic amino acid tryptophan ^[44]^. Gut-derived indole and its derivatives can regulate intestinal barrier integrity, immunity, and gut hormones to impact hepatic inflammation and energy metabolism ^[45]^. Oral administration of indole in mice decreased LPS-induced liver inflammation by reducing hepatic pro-inflammatory gene expression (IL-1b, IL-6, and IL-15) and ameliorated oxidative stress by downregulating the expression genes *Nos2* (nitric oxide synthase 2) and *Nox2* (NADPH oxidase 2) ^[46]^. Ex vivo experiments using the model of precision-cut liver slices demonstrated that the anti-inflammatory effect of indole is mediated by the suppression of nucleotide-binding domain and leucine-rich repeat–containing family pyrin domain–containing 3 (NLRP3) signaling pathway ^[46]^.

Indole-3-acetic acid (IAA) is another gut microbiota-derived metabolite from dietary tryptophan. Intraperitoneal injection of IAA at a dose of 50 mg/kg bodyweight in mice alleviated HFD-induced insulin resistance, high fasting blood glucose levels, and liver total TGs and cholesterol ^[47]^. In addition, IAA treatment decreased hepatic inflammation and oxidative stress by reducing F4/80^+^ macrophage infiltration, the expression of TNF-α and MCP-1/CCL2, and the production of reactive oxygen species, malonaldehyde, and glutathione, as well as the SOD activity in liver tissues ^[47]^.

Metabolites tryptamine and indole-3-acetate (I3A) are produced by gut microbiota, which are absent from germ-free mice and can be deprived by HFD treatment. Treatment with both metabolites can decrease fatty acid (palmitic and oleic acids)- and LPS-stimulated expressions of pro-inflammatory cytokines in macrophages and inhibit chemokine (CCL2)-induced macrophage migration. In addition, I3A treatment can reduce fatty acid and/or TNF-α–induced hepatocyte inflammation by decreasing the expression of fatty acid synthase and sterol regulatory element-binding protein-1c ^[48]^. In summary, indole and tryptophan-derived metabolites can suppress liver inflammation.

### 3.3 Linoleic acid (LA)

Oral treatment of *Lactobacillus reuteri* reversed ethanol-induced hepatitis, inflammatory cell infiltration, and lipid accumulation through the regulation of fatty acid metabolic pathways, such as alpha-linolenic acid and linoleic acid (LA) metabolism pathways ^[49]^. Bacteria such as *Lactobacillus* and *Bifidobacterium* can convert LA to conjugated linoleic acid (CLA) ^[50,51]^. Treatment of CLA significantly decreased the mRNA expression of liver TNF-α, IFN-γ, and IL-1β and increased the mRNA and protein expression of intestinal tight junction proteins (occludin and ZO-1) in ob/ob (obese) mice ^[52]^. In addition, CLA treatment in these mice increased the abundance of beneficial bacteria, such as *Lachnoclostridium*, *Roseburia*, *Dubosiella*, *Oscillibacter*, and *Anaerostipes*, and decreased the abundance of pro-inflammatory bacteria, such as *Tyzzerella* and *Alistipes*. However, CLA treatment displayed different functions in wild-type mice, which can induce hepatic inflammation and increase intestinal permeability ^[52]^. Another study also showed that pregnant rats with an LA-rich diet predispose offspring to develop hepatic steatosis and subsequent metabolic disorders ^[53]^. The underlying cellular and molecular mechanisms of LA- and CLA-mediated effects in liver inflammation need to be further studied.

### 3.4 Phenylacetic acid (PAA)

Phenylacetic acid (PAA) is a gut microbiota-dependent metabolic derivative of dietary phenylalanine, produced mainly by three gut microbiota phyla, including Bacteroidetes, Firmicutes, and Proteobacteria ^[54]^. PAA can be further conjugated to glutamine to form phenylacetylglutamine (PAGln) in primates or to glycine to form phenylacetylglycine in rodents. PAGln has been shown to be positively associated with CVD and its associated morbidity and mortality by interacting with G-protein coupled receptors, including α2A, α2B, and β2-adrenergic receptors ^[55]^.

### 3.5 Pentadecanoic acid

The increase of inulin-induced commensal *Parabacteroides distasonis* can increase the production of pentadecanoic acid, restore gut barrier integrity, and suppress hepatic steatosis and inflammation by decreasing serum levels of LPS and hepatic pro-inflammatory cytokines (eg, TNF-α and IL-6) in mouse NASH models ^[56]^. Another study revealed that treatment with a combination of live *Bifidobacterium*, *Lactobacillus*, *Enterococcus*, and *Bacillus* can ameliorate cyclophosphamide-induced rat death, weight loss, and gut, liver, spleen, and lungs damage by regulating gut microbiota profiles, including an increase in phylum levels of Proteobacteria, Fusobacteriota, and Actinobacteriota and a decrease of Spirochaetota and Cyanobacteria ^[57]^.

### 3.6 Short-chain fatty acids (SCFAs)

C57BL/6 mice fed with a diet containing 5% ethanol had increased hepatic fat accumulation and serum markers of liver injury, such as ALT and AST. This feeding alcohol diet caused intestinal barrier disruption and gut dysbiosis with an increase in the relative abundance of pathogenic bacteria *Escherichia* and *Staphylococcus* and depletion of SCFA-producing bacteria, including *Prevotella, Faecalibacterium*, and *Clostridium* ^[31]^. In contrast, administration of *P. pentosaceus*, an ethanol-resistant probiotic strain, increased the production of SCFAs (propionic acid and butyric acid) by inhibiting an alcohol-induced decrease in the relative abundance of gut microbiota *Lactobacillus*, *Pediococcus*, *Prevotella*, *Clostridium*, and *Akkermansia* ^[31]^. This protective effect also promoted the expression of intestinal tight junction proteins, mucins, and antimicrobial peptides ^[31]^.

Supplementation of *Bifidobacterium breve* and *Bifidobacterium longum* ameliorated the progression of NAFLD in mice by reducing the *Firmicutes*/*Bacteroidetes* ratio in the gut microbial profiles and increasing the production of SCFAs (acetic acid, butyric acid, and propionic acid) and tryptophan metabolites (eg, indole-3-propionic acid and -acrylic acid) ^[58]^.

However, SCFAs may regulate immune responses to increase the development of HCC. Feeding a fermentable fiber diet can cause silent portosystemic shunt and cholemia in mice by increasing the production of SCFAs and BAs, predisposing liver injury and progression of cholestatic HCC ^[59]^. Studies also found that the levels of SCFAs (eg, butyrate) and their intermediates were increased in the feces and sera of patients with NAFLD-HCC compared with patients with NAFLD-cirrhosis and non-NAFLD controls ^[60]^. Mechanistically, the bacterial extract from patients with NAFLD-HCC compared with that from patients with NAFLD-cirrhosis and non-NAFLD controls significantly increased the frequency of Tregs but decreased the frequencies of CD8^+^ T cells, CD14^+^ monocytes, and CD19^+^CD20^+^ B cells in peripheral blood mononuclear cells from non-NAFLD controls ^[60]^.

### 3.7 Secondary bile acids (SBAs)

SBAs are produced by gut microbiota from PBAs that are synthesized in the liver ^[61]^. Oral gavage of live *A. muciniphila* can decrease the production of SBAs in the cecum, such as deoxycholic acid (DCA) and lithocholic acid (LCA), to ameliorate hepatic steatosis and inflammation in mice with HFD-induced MAFLD ^[26]^. The serum levels of SBAs were positively and significantly associated with liver fibrosis in patients with histologically approved NAFLD, especially for patients with mild fibrosis ^[62]^. Blocking DCA production or decreasing gut microbiota that produces DCA can prevent HCC development in obese mice by reducing senescence-associated secretory phenotype in hepatic stellate cells ^[63]^. BAs such as chenodeoxycholic acid (a PBA) and DCA (a SBA) can induce IL-1α and IL-1β secretion in LPS-primed one marrow-derived dendritic cells in vitro and regulate the migration of neutrophils and monocytes in vivo ^[64]^.

### 3.8 Taurocholic acid

The gut microbiota composition in mice with a high-cholesterol diet changed during the development of hepatic steatosis, steatohepatitis, and HCC, including a sequential increase of *Mucispirillum*, *Desulfovibrio*, *Anaerotruncus*, and *Desulfovibrionaceae* and a depletion of *Bifidobacterium* and *Bacteroides*. This phenomenon was also shown in human patients with hypercholesterolemia ^[65]^. Gut bacterial metabolites taurocholic acid and 3-indolepropionic acid were increased and decreased in these mice, respectively.

### 3.9 Trimethylamine N-oxide (TMAO)

Trimethylamine *N*-oxide (TMAO) is a metabolite of dietary choline, betaine, or carnitine generated by gut microbiota, which can aggravate hepatic TG accumulation in mice and increase lipogenesis in palmitic acid-treated HepG2 cells. TMAO-induced lipid production is mediated by upregulation of the synthesis of BAs that can activate the Farnesoid X receptor signaling pathway ^[66]^.

Overall, gut microbiota-derived metabolites can impact liver inflammation and disease progression (Table 2). Treatments that increase the favorable gut microbiota or suppress the unfavorable bacteria can inhibit liver inflammation, and vice versa.

**Table 2:** Gut microbiota-derived metabolites in liver disease and inflammation.

Liver disease	Metabolite	Function	References
Non-alcoholic steatohepatitis (NASH)	Ethanol	Endogenous ethanol is positively associated with the development of NASH in human patients with an increased abundance of alcohol-producing bacteria, such as phylum Proteobacteria, family *Enterobacteriaceae*, and genus *Escherichia*.	Zhu et al ^[40]^
Liver inflammation	Indole	Suppress LPS-induced liver inflammation by inhibiting nucleotide-binding domain and leucine-rich repeat–containing family pyrin domain–containing 3 (NLRP3) pathway.	Beaumont et al ^[46]^
Non-alcoholic fatty liver disease (NAFLD)	Indole-3-acetic acid (IAA)	Decrease hepatic inflammation and oxidative stress by reducing F4/80^+^ macrophage infiltration, expression of TNF-α and monocyte chemoattractant protein-1 (MCP-1 or CCL2), and production of reactive oxygen species, malonaldehyde, and glutathione, as well as the superoxide dismutase activity in liver tissues.	Ji et al ^[47]^
Hepatocyte inflammation and steatosis	Indole-3-acetate (I3A)	I3A treatment can reduce fatty acid and/or TNF-α–induced hepatocyte inflammation by decreasing the expression of fatty acid synthase (FAS) and sterol regulatory element-binding protein-1c (SREBP-1c).	Krishnan et al ^[48]^
Alcoholic liver disease (ALD)	Alpha-linolenic acid and linoleic acid	Oral treatment of *Lactobacillus reuteri* reversed ethanol-induced hepatitis, inflammatory cell infiltration, and lipid accumulation through regulation of fatty acid metabolic pathways, such as alpha-linolenic acid and linoleic acid metabolism pathways.	Zheng et al ^[49]^
NASH	Pentadecanoic acid	The increase of commensal *Parabacteroides distasonis* induced by inulin can increase the production of pentadecanoic acid and restore gut barrier integrity and hepatic steatosis and inflammation in mouse NASH models.	Wei et al ^[56]^
Metabolic-associated fatty liver disease (MAFLD)	Secondary bile acids (SBAs) such as deoxycholic acid (DCA) and lithocholic acid (LCA).	Oral gavage of live *A. muciniphila* can decrease the production of SBA in the cecum, such as DCA and LCA to ameliorate hepatic steatosis and inflammation in mice with HFD-induced MAFLD.	Wu et al ^[26]^
NAFLD and mild liver fibrosis	SBAs	The serum levels of SBAs were positively and significantly associated with liver fibrosis in patients with NAFLD, especially for NAFLD patients with mild liver fibrosis.	Liu et al ^[62]^
Hepatic steatosis or lipid accumulation	Trimethylamine *N*-oxide (TMAO)	TMAO can aggravate hepatic triglyceride accumulation in mice and increase lipogenesis in palmitic acid (PA)-treated HepG2 cells, which can upregulate the synthesis of bile acids.	Tan et al ^[66]^

NASH, non-alcoholic steatohepatitis; LPS, lipopolysaccharide.

## 4. Factors regulate the change of gut microbiota and their metabolites to impact liver inflammation

Factors including lifestyle, environment, medicines, hormones, and genetics impact gut microbiota profiles and their metabolites to aggregate or dampen hepatic inflammation and injury. In this section, we discuss the latest updates on how these factors impact gut microbial profiles and liver inflammation.

### 4.1 Diet and drink

Energy (eg, lipid and sugar) metabolism can significantly impact the profiles of gut microbiota and their metabolites. For example, alterations in glucose, fatty acid, and lipoprotein metabolism are commonly associated with NAFLD and obesity in mice and humans ^[67]^, resulting in systemic and local liver inflammation. Insulin plays an important role in the regulation of energy metabolism in tissues such as adipose tissue, liver, and intestine, which can be impacted by gut microbiota and their metabolites ^[68,69]^. Insulin resistance increases the circulating levels of glucose and drives hepatic de novo lipogenesis to aggregate NAFLD progression ^[70]^.

Excessive alcohol intake ^[71,72]^, high-fat and/or high-sugars diets ^[73,74]^, contaminated food ^[75,76]^, and inappropriately cooked food ^[77]^ can cause changes of gut microbial profile, interruption of metabolic homeostasis, and liver injury. Serum levels of LPS, expression of pro-inflammatory cytokines and chemokines (eg, IL-1β and CCL2), hepatocyte injury, and infiltration of circulating inflammatory cells (eg, neutrophils and macrophages) are commonly associated with diet-induced gut microbiota dysbiosis and liver inflammation ^[72,78]^. As discussed above, an increase of alcohol-producing bacteria (eg, *Enterobacteriaceae*) can accelerate liver inflammation, whereas lipid-digesting and mucin-degrading bacteria (eg, *A. muciniphila*) may reduce HFD-induced hepatic inflammation and steatosis.

### 4.2 Exercise

Exercise can reduce obesity, metabolic syndrome development, and hepatic steatosis in HFD-fed rats. Exercise further improves the homeostasis of BA synthesis and reverses diet-induced imbalance of gut microbiota by increasing gut microbial genera *Parabacteroides*, *Bacteroides*, and *Flavobacterium* and decreasing genera *Blautia*, *Dysgonomonas*, and *Porphyromonas* ^[79]^. Aerobic exercise can also restore intestinal tight junctions, suppress LPS production and LPS-binding protein expression, and reduce liver inflammation by inhibiting LPS/TLR4/nuclear factor (NF)-κB signaling pathway ^[80]^. In patients with NAFLD, exercise can significantly reduce body mass index (BMI), plasma TGs and apolipoprotein B, visceral fat area, body fat mass, and intrahepatic lipid content, when comparing responders with non-responders post 12-week intervention. One of the underlying mechanisms is that exercise significantly restructures the gut bacteria interactome, which benefits metabolic balance ^[81]^. In addition to the improvement of hepatic fat accumulation and metabolism, exercise can also regulate the function of liver resident macrophages to modulate liver inflammation and fibrosis ^[82]^. In addition, exercise can improve mitochondrial function to enhance aerobic metabolism ^[83]^. However, exercise can also directly cause abnormal liver function tests in healthy young adults ^[84]^. For example, strenuous exercise (vigorous exercise) increases hepatocyte permeability inducing an upregulation of liver enzymes ^[84]^.

### 4.3 Socioeconomic and environmental factors

Socioeconomic factors such as income, education, employment, and social communication can impact the profiles of gut microbiota ^[85,86]^. One study showed that there was a significant association between the number of socioeconomic parameters and prediction of NASH (four or more) or severe steatosis (six or more), but not advanced fibrosis ^[87]^. These socioeconomic factors include employment status, education degree, percentage of foreign born, private vs public health care, percentage without a car, liver environment, and so on. Another study also showed that the incidence rate of ALD was negatively correlated with the education level ^[88]^. In relation, air pollution has been shown to impact the composition of gut microbiota in rodent models and metabolic signaling pathways ^[89–91]^, which may impact liver disease. For example, a study showed that long-term exposure to NO_2_ impacts liver function in patients with schizophrenia by significantly increasing the levels of gamma-glutamyl transpeptidase and glutamic pyruvic transaminase ^[92]^. Gut microbial phyla including Firmicutes, Actinobacteria, and Proteobacteria played an intermediary role in this process ^[92]^.

### 4.4 Drugs

Antibiotics are the most common treatments for bacterial infections. The “double-edged sword” effect of antibiotic treatment with resultant gut microbiota changes has been illustrated in the last century ^[93]^. For example, treatment with an antibiotic cocktail consisting of vancomycin, neomycin, and primaxin alters gut commensal bacteria, which can suppress HCC development via regulation of hepatic NKT cell function and IFN-γ production upon antigen stimulation ^[23]^. In contrast, gut microbiota plays an important role in drug-induced hepatotoxicity. Treatment with the anti-cancer drug cisplatin can induce liver inflammation and cell apoptosis while also increasing the relative abundance of gut microbiota, such as *Escherichia*, *Parabacteroides*, and *Ruminococcus*. In addition, ablation of gut microbiota by antibiotics can protect against anti-cancer drug cisplatin-induced liver cytotoxicity ^[94]^. Meanwhile, studies also show that antibiotic treatment can compromise immune checkpoint inhibitor efficacy, correlating with lower objective response rates, overall survival, and progression-free survival ^[95]^.

### 4.5 Hormones

Melatonin, a hormone produced in the brain in response to darkness, can reduce aflatoxin B1–induced liver injury via the suppression of TLR4, MyD88, phospho-NF-κB (p-p56), and p-IκBα (nuclear factor of kappa light polypeptide gene enhancer in B cells inhibitor-alpha) ^[76]^. However, melatonin may lose its function in mice treated with antibiotics ^[76]^. Another study showed that melatonin treatment significantly reduced the relative abundance of gut bacterial genera *Lactobacillus* and *Desulfovibrio* and increased the abundance of genera *Bacteroides*, ameliorating ochratoxin A–induced liver inflammation and restoring gut barrier function ^[96]^.

### 4.6 Genetic factors

Sirtuin 2 (SIRT2) deficiency promoted lipid deposition and inflammation in cells accompanied with HFCS (high-fat/high-cholesterol/high-sucrose)-induced obesity, hepatic steatosis, and an aggravated metabolic profile, indicating SIRT2 deficiency advances NAFLD–NASH progression. Further study indicated that SIRT2 deficiency induced gut microbiota dysbiosis, inducing a decrease of bacterial genera *Bacteroides* and *Eubacterium* and an increase of genus *Acetatifactor* compared with the gut microbial profiles in wild-type mice ^[97]^. In addition, the profiles of serum metabolites changed in mice with SIRT2 knockout, with an upregulation of l-proline and downregulation of phosphatidylcholines, lysophosphatidylcholine, and epinephrine. The expression of SIRT2 was downregulated in human patients with NALFD compared with healthy controls, accompanying the progression of NAFLD to NASH ^[97]^. Another study showed that SIRT1 deficiency in the intestine in mice with bile duct ligation can cause intestinal inflammation by increasing macrophage infiltration and cytokine expression (eg, TNF-α and IL-6) while limiting the production of SCFAs ^[98]^.

Overall, given the important roles of gut microbiota-derived metabolites in systemic and liver inflammation, manipulations of gut microbiota with different strategies are potential options for the suppression of inflammation, both locally and systematically.

## 5. Clinical trials

Currently, many clinical trials are evaluating the efficacy of different treatments involving the gut–liver axis, including FMT ^[99,100]^, drugs (eg, anti-alcoholism drug disulfiram) ^[101]^, diet ^[102]^, probiotics ^[103–106]^, synbiotics ^[107]^, antibiotics ^[108]^, fibroblast growth factor 19 analog (aldafermin or NGM282) ^[109]^, and physical activity ^[110]^. For example, allogenic FMT from a healthy donor to NAFLD patients significantly improved intestinal permeability after 6 weeks compared to baseline ^[100]^. Aldafermin treatment significantly enriched the gut microbial genus *Veillonella* in a dose-dependent manner, which may reduce toxic BAs in patients with NASH ^[109]^. A 3-month physical activity intervention with an inulin-enriched diet decreased BMI, liver enzymes, and plasma cholesterol and improved glucose tolerance in subjects with obesity. In addition, physical exercise significantly increased inulin-induced regulation of *Bifidobacterium*, *Dialister*, and *Catenibacterium* genera ^[110]^. Oral administration of pasteurized *A. muciniphila* significantly reduced insulinemia, improved insulin sensitivity, and reduced plasma total cholesterol in patients with obesity-related metabolic disorders compared to placebo. Furthermore, pasteurized *A. muciniphila* treatment was associated with decreased body weight, fat mass, and hip circumference compared to baseline ^[111]^. In patients with cirrhosis, antibiotic rifaximin-α treatment improved hepatic encephalopathy, reduced systemic inflammation, and repaired intestinal barrier via reducing levels of mucin-degrading sialidase-rich species, such as *Streptococcus* spp, *Veillonella atypica* and *Veillonella parvula*, *Akkermansia*, and *Hungatella* ^[108]^.

In addition, there are many recruiting clinical trials that aim to evaluate the effects of gut microbiota regulation on liver diseases (Clinicaltrials.gov, such as NCT05006430 and NCT04932577) (Table 3). However, some treatments do not show promising effects. For example, the use of probiotic supplements (*Lactobacillus acidophilus* ATCC SD5221 and *Bifidobacterium lactis* HN019) alone for 6 months only improved the AST to platelet ratio index score in patients with NASH but did not change liver enzymatic markers, inflammatory parameters, hepatic steatosis and fibrosis, and gut microbiota significantly ^[105]^. Another clinical trial (NCT03127696) showed that repeated FMTs can increase the level of microbiota engraftment and its duration in patients with obesity and type 2 diabetes. In addition, FMT plus lifestyle modification can increase beneficial microbiota in recipients to improve lipid metabolism and liver stiffness ^[112]^. Furthermore, a mixed probiotic treatment (NCT04074889) with multiple strains containing six different *Lactobacillus* and *Bifidobacterium* species can improve mucosal immune function to reduce intestinal permeability in patients with NAFLD ^[103]^. Overall, these results suggest that synergistic treatments may improve the efficacy of therapeutics.

**Table 3:** Clinical trials of modulating gut microbiota to treat liver inflammation and chronic disease.

Trial number	Phase	Treatment	Functions	References
NCT02496390	1 or 2	FMT	Allogenic FMT from a thin and healthy donor to NAFLD patients significantly improved the intestinal permeability after 6-week treatment compared to baseline.	Craven et al ^[100]^
NCT02972567	N/A	*Lactobacillus reuteri* V3401	The impact of probiotic treatment (*L. reuteri* V3401) on the change of plasma lipopolysaccharide (LPS) levels, anthropometric parameters, lipid profile, glucose metabolism, microbiota composition, hepatic steatosis, inflammation, and cardiovascular biomarkers.	Tenorio-Jiménez et al ^[104]^
NCT02764047	N/A	Probiotic supplement (*Lactobacillus acidophilus* ATCC SD5221 and *Bifidobacterium lactis* HN019)	Probiotic supplementation alone for 6 months improved the aspartate aminotransferase (AST) to platelet ratio index (APRI) score, but not liver enzymatic markers, inflammatory parameters, and gut microbiota in patients with NASH.	Escouto et al ^[105]^
NCT04787276	N/A	*Escherichia coli* Nissle (EcN) 1917 strain	Probiotic treatment can decrease the serum levels of pro-inflammatory cytokines, normalize gut microbiota composition, and improve the cognitive function of patients with hepatic encephalopathy.	Manzhalii et al ^[106]^
NCT01680640	N/A	Synbiotic	Administration of a synbiotic combination (probiotic and prebiotic) for a 1 year changed fecal microbiota but did not decrease hepatic fat content and fibrotic markers.	Scorletti et al ^[107]^
NCT02019784	4	Rifaximin-α	Leading to hepatic encephalopathy in patients with cirrhosis, systemic inflammation, and repaired intestinal barrier via reducing levels of mucin-degrading sialidase-rich species, such as *Streptococcus* spp, *Veillonella atypica* and *Veillonella parvula*, *Akkermansia*, and *Hungatella*.	Patel et al ^[108]^
NCT02443116	2	Aldafermin or NGM282	Aldafermin treatment significantly enriched the gut microbial genus *Veillonella* in a dose-dependent manner, which might reduce the toxic bile acids in patients with NASH.	Loomba et al ^[109]^
NCT03852069	N/A	Physical activity	A 3-month physical activity intervention plus an inulin-enriched diet decreased BMI, liver enzymes, and plasma cholesterol, and improved glucose tolerance in subjects with obesity, and more physical exercise can significantly increase inulin-induced regulation of microbial genera *Bifidobacterium*, *Dialister*, and *Catenibacterium*.	Rodriguez et al ^[110]^
NCT02637115	N/A	*Akkermansia muciniphila*	Reducing insulinemia and plasma total cholesterol and improving insulin sensitivity in patients with obesity-related metabolic disorders.	Depommier et al ^[111]^
NCT03127696	N/A	FMT	Repeated FMTs can increase the level of microbiota engraftment and its duration in patients with obesity and type 2 diabetes. In addition, FMT plus lifestyle modification can increase beneficial microbiota in recipients to improve lipid metabolism and liver stiffness.	Ng et al ^[112]^
NCT04074889	N/A	Six different *Lactobacillus* and *Bifidobacterium* species	This mixed probiotic can improve mucosal immune function to reduce intestinal permeability in patients with NAFLD.	Mohamad Nor et al ^[103]^

Aldafermin, fibroblast growth factor 19 analog; BMI, body mass index; FMT, fecal microbiota transplantation; N/A, not applicable; NAFLD, non-alcoholic fatty liver disease; NASH, non-alcoholic steatohepatitis.

## 6. Conclusions

**Figure 1.**
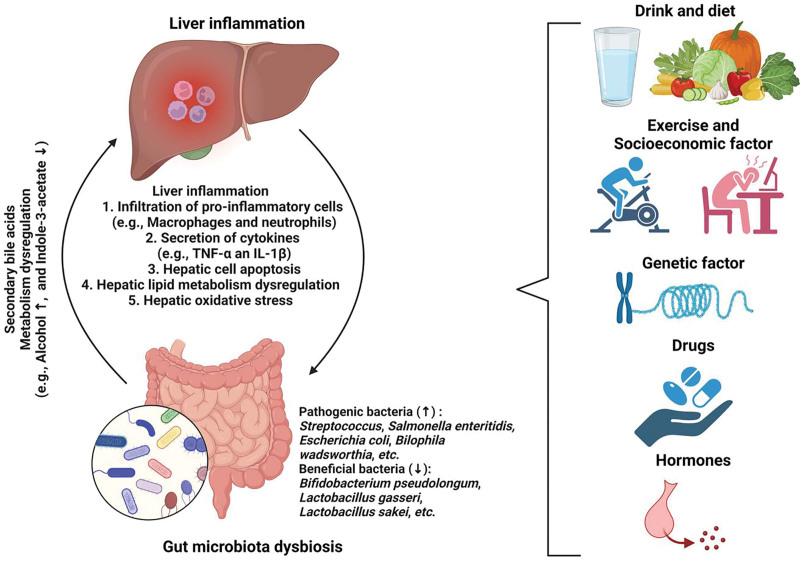
**The roles of gut microbiota and metabolites in liver inflammation and potential regulatory factor.** Gut microbiota dysbiosis impacts liver inflammation, accompanying the infiltration of pro-inflammatory cells (eg, macrophages and neutrophils), secretion of inflammatory cytokines (eg, TNF-α, IL-1β, and IFN-γ), hepatic cell apoptosis, lipid accumulation, and oxidative stress. Alteration of gut microbiota including the increase of pathogenic bacteria and decrease of beneficial bacteria can change the products of secondary bile acids and metabolites such as alcohol and indole metabolism to promote liver inflammation. Factors including diet, drink, exercise, stress, genetic factors, drugs, and hormones can regulate the change of gut microbiota to treat liver inflammation. IFN-γ, interferon-γ; IL-1β, interleukin-1β; TNF-α, tumor necrosis factor-α. The figure was prepared using Biorender (https://biorender.com).

Liver inflammation is commonly associated with metabolic liver diseases, such as ALD, NAFLD or MAFLD, liver fibrosis, cirrhosis, and cancers. Accumulating evidence shows that gut microbiota can impact liver inflammation in various liver diseases through the gut–liver axis, the physiological crosstalk between the gut and liver via immunological and metabolic signals. This axis can be regulated by dietary, genetic, and environmental factors. Factors including lifestyle, environment, medicines, hormones, and genetics can impact the gut microbial profiles and their metabolites to aggregate or dampen liver inflammation and injury (Figure 1). Clinical trials have been performed or are recruiting to test the application of FMT, drugs (eg, anti-alcoholism drug disulfiram), diets, probiotics, synbiotics, antibiotics, fibroblast growth factor 19 analog (aldafermin or NGM282), and physical activity for the treatment of liver inflammation and gut microbiota dysbiosis. Current clinical trials show that a single treatment alone (eg, probiotics) cannot significantly reduce liver inflammation by regulating the gut–liver axis. Synergistic treatment or a combined treatment of microbial cocktail may be an option to improve the treatment efficacy via regulating gut microbial profile to suppress liver disease progression. Meanwhile, the underlying mechanisms causing the failure of the treatments in patients remain to be clarified.

## Author contributions

Design, writing original draft: M.Y.; review and editing: M.Y., K.M., E.T.K., K.F.S.-O., and G.L.; supervision: E.T.K., K.F.S.-O., and G.L.; funding acquisition: E.T.K., K.F.S.-O., and G.L. All authors have read and approved the manuscript.

## Conflicts of interest

The authors declare no conflict of interest.

## Funding

The authors were funded by grants from NIH R01DK130340 (G.L., K.F.S.-O., R.S.R.), NIH R01CA208396 (G.L., M.K., K.F.S.-O.), NIH R01CA250536 (G.L. and K.F.S.-O.), and NIH R01CA274959 (G.L. and K.F.S.-O.); partly supported by VA Merit Award I01 BX004065-1 (E.T.K. and K.F.S.-O.).
